# Fatty Acid Levels and Their Inflammatory Metabolites Are Associated with the Nondipping Status and Risk of Obstructive Sleep Apnea Syndrome in Stroke Patients

**DOI:** 10.3390/biomedicines10092200

**Published:** 2022-09-06

**Authors:** Arleta Drozd, Dariusz Kotlęga, Przemysław Nowacki, Sylwester Ciećwież, Tomasz Trochanowski, Małgorzata Szczuko

**Affiliations:** 1 Department of Human Nutrition and Metabolomics, Pomeranian Medical University in Szczecin, 71-460 Szczecin, Poland; 2 Department of Pharmacology and Toxicology, University of Zielona Góra, 65-001 Zielona Góra, Poland; 3 Department of Neurology, Pomeranian Medical University in Szczecin, 71-252 Szczecin, Poland; 4 Department of Gynecology, Endocrinology and Gynecologic Oncology, Pomeranian Medical University in Szczecin, 71-252 Szczecin, Poland; 5 Department of Neurological Rehabilitation, District Hospital, 67-200 Głogów, Poland

**Keywords:** dipping, inflammation, eicosanoids, cardiovascular disease, leukotriene, saturated fatty acids

## Abstract

Background: This paper discusses the role of inflammation in the pathogenesis of nondipping blood pressure and its role in the pathogenesis of obstructive sleep apnea syndrome. The aim of the study was to assess the impact of free fatty acids (FAs) and their inflammatory metabolites on the nondipping phenomenon and the risk of sleep apnea in stroke patients. Methods: Sixty-four ischemic stroke patients were included in the prospective study. Group I consisted of 33 patients with a preserved physiological dipping effect (DIP), while group II included 31 patients with the nondipping phenomenon (NDIP). All subjects had FA gas chromatography and inflammatory metabolite measurements performed with the use of liquid chromatography, their 24 h blood pressure was recorded, and they were assessed with the Epworth sleepiness scale (ESS). Results: In the nondipping group a higher level of C16:0 palmitic acid was observed, while lower levels were observed in regard to C20:0 arachidic acid, C22:0 behenic acid and C24:1 nervonic acid. A decreased leukotriene B4 level was recorded in the nondipping group. None of the FAs and derivatives correlated with the ESS scale in the group of patients after stroke. Correlations were observed after dividing into the DIP and NDIP groups. In the DIP group, a higher score of ESS was correlated with numerous FAs and derivatives. Inflammation of a lower degree and a higher level of anti-inflammatory mediators from EPA and DHA acids favored the occurrence of the DIP. A high level of C18: 3n6 gamma linoleic acid indicating advanced inflammation, intensified the NDIP effect. Conclusions: We demonstrated potential novel associations between the FA levels and eicosanoids in the pathogenesis of the nondipping phenomenon. There are common connections between fatty acids, their metabolites, inflammation, obstructive sleep apnea syndrome and nondipping in stroke patients.

## 1. Introduction

### 1.1. Fatty Acids and Stroke

Arterial stiffness increases with age due to atherosclerosis, arteriosclerosis, and calcifications of the vessel. Vascular calcifications, resulting from nucleation of calcium and phosphate into crystals, is associated with a transformation of vascular smooth muscle cells into osteoblast-like cells [1]. Polyunsaturated fatty acids (PUFA) are recommended by the guidelines of the European Society of Cardiology to reduce blood pressure, low density lipoprotein (LDL) cholesterol, the synthesis of proinflammatory mediators, and to increase the availability of nitric oxide in the vascular wall [1,2]. Prevalent vegetable oil users, especially extra-virgin olive oil and corn oil, had lower arterial stiffness compared to prevalent animal fat users in a study [3].

Serum fatty acid levels are associated with the risk of ischemic stroke (IS), however, the effect of saturated and unsaturated fatty acids on the risk of IS remains uncertain and the available results are inconsistent [4,5]. The role of n-3 and n-6 polyunsaturated fatty acids is also equivocal [6,7]. In the Framingham Study population, serum palmitic acid (C16:0) level, which is a saturated fatty acid (SFA), increased the risk of ischemic stroke (HR 1.76; 9% CI: 1.26–2.45) [6]. On the other hand, in the Atherosclerosis Risk in Communities (ARIC) Study, palmitic acid level was not found to be connected with the risk of IS [8]. The long chain n-3 polyunsaturated fatty acids (n-3 PUFA) which play an important role in cardiovascular risk include: eicosapentaenoic acid (EPA), docosahexaenoic acid (DHA) and docosapentaenoic acid (DPA). They decrease the risk of hypertension, dyslipidemia, endothelial dysfunction and thus they are supposed to have a protective impact on cardiovascular diseases [9]. The level of n-3 polyunsaturated fatty acids such as α-linolenic acid (C18:3n3; ALA) was not shown to affect the risk of ischemic stroke, while in another study such a relationship was confirmed [10,11]. In a meta-analysis from the year 2012, n-3 PUFA supplementation was not associated with a lower risk of stroke, but in a recent meta-analysis from 2018 the analyzed fish intake was reported to lower the risk of stroke [12,13]. Marine omega-3 PUFAs may protect against ventricular arrhythmias, and there is growing evidence for an effect of marine omega-3 PUFAs in the prevention and treatment of atrial fibrillation [14]. In a prospective study with a follow-up of 8.3 to 11.2 years, higher levels of circulating DPA and DHA were connected with a lower risk of IS [9]. Moreover, trans unsaturated fatty acids (from margarine, cakes, and pastries), are not recommended by the European and American guidelines. They impair postprandial vascular endothelial function but not arterial stiffness in humans [15].

How monounsaturated fatty acids (MUFA) influence the risk of stroke is yet to be clarified, as for the time being there is still limited data available in this area of research [16,17].

### 1.2. The Eicosanoids

Ischemic stroke is associated with the presence of a strong inflammatory reaction, in which arachidonic acid (AA) plays a key role. Derivatives are synthesized using three lipoxygenase pathways—5LOX, 15LOX and cyclooxygenase COX1 and COX2. It seems that 12LOX is not involved in this process [18].

The leukotrienes (LT) are lipid mediators belonging to a large family of molecules named eicosanoids. They are generated from arachidonic acid (AA), a carbon-20 PUFA, through the 5-lipoxygenase (5-LOX) pathway. Cysteinyl leukotrienes (CysLTs) are potent lipid inflammatory mediators and play a crucial role in the pathogenesis of inflammation. Therefore, CysLT modifiers as synthesis inhibitors or receptor antagonists, may become a potential target for the treatment of other inflammatory diseases such as cardiovascular disorders. The cardioprotective effects observed by using CysLT modifiers are promising and contribute to elucidate the link between CysLTs and cardiovascular disease [19]. Chronic, low-grade inflammation has been involved in the pathogenesis of atherosclerosis. A specific group with an increased risk of cardiovascular diseases are women with PCOS who would also be included in clinical trials [20].

### 1.3. The Nondipping Blood Pressure Phenomenon and Inflammation

Blood pressure fluctuates following the circadian rhythm, which originates in the suprachiasmatic nuclei of the anterior hypothalamus. Nocturnal dipping of blood pressure mainly results from this physiological rhythm, and a lack of dipping is associated with increased cardiovascular disorders and more severe end-organ damage [21,22]. The cause of the nondipping phenomenon lies in the inability to modulate autonomic tone [23]. The 24 h monitoring of blood pressure is performed with the use of so-called ambulatory blood pressure monitoring (ABPM). The physiological nocturnal reduction in both systolic and diastolic blood pressure should range from 10% to 20%, defining individuals as dippers, while subjects that do not reach 10% are referred to as nondippers [24,25]. A nighttime blood pressure value in hypertensive patients is an independent risk factor of cardiovascular disease, defined as stroke or coronary artery disease [26]. Nondippers were found to have a significantly higher risk for cerebral infarction (RR 1.59 (95% CI, 1.03 to 2.46); *p* = 0.04), regardless of the use of antihypertensives [27]. The nondipping status plays an important role in the cardiovascular risk also in subjects without hypertension. The nondipping pattern in 24 h blood pressure values within the normal range was connected with an increased risk of cardiovascular mortality and greater organ damage [28].

The role of inflammation in the pathogenesis of the nondipping pattern is still being analyzed. The inflammatory markers were found to be elevated in hypertensive individuals and to correlate with the risk of stroke [29,30]. An increased level of high-sensitivity C-reactive protein (hsCRP) was detected in nondipping patients with obstructive sleep apnea syndrome (OSAS) and with newly diagnosed hypertension [31,32]. OSAS is a risk factor for cardiovascular episodes, including ischemic stroke. The frequency of the nondipping pattern in OSAS patients reaches up to 84% and increases with OSAS severity. The risk of the nondipping pattern is 1.5 times higher in OSAS patients, as reported by a recent meta-analysis [33]. The risk of OSAS may be assessed with the use of the Epworth sleepiness scale (ESS), whose sensitivity reaches up to 76% [34]. Fatty acids play a role in the inflammatory process. The main anti-inflammatory effect is ascribed to n3 fatty acids, especially DHA [35]. A role in proinflammatory reactions is associated with n-6 PUFA—arachidonic acid (AA) [36]. Several fatty acids, including n-3 and n-6, are associated with inflammatory markers in subjects with high cardiovascular risk [37].

There are limited available data regarding the association between dipping status, serum fatty acids and their inflammatory metabolites, either in the general population or in ischemic stroke patients. There is also limited information about the role of nondipping blood pressure in the pathogenesis of stroke. The inflammatory pathogenetical aspects of OSAS were taken into consideration by assessing its risk with the use of ESS.

## 2. Materials and Methods

### 2.1. Study Design and Population

The study included 64 ischemic stroke patients who were hospitalized in the Neurology Department in the district hospital in Poland. All subjects were Caucasians, and all patients were treated with statins and acetylsalicylic acid, and previous hypotensive treatment was maintained. Group I consisted of 33 patients with preserved physiological dipping effect (DIP), while group II included 31 patients with the nondipping phenomenon (NDIP). The inclusion criterion was the detection of ischemic stroke on the basis of clinical symptoms and additional test results, with standard treatment [38,39]. Informed consent was obtained from all the patients.

The exclusion criteria included intracranial hemorrhage visible on brain imaging, speech or consciousness disturbances which would make obtaining the informed consent impossible, presence of active infection including body temperature of more than 37.4 °C, clinical or biochemical symptoms of infection, active autoimmune disorder or malignancy. The fasting blood samples were taken on the seventh day of hospitalization. The characteristics of both groups are presented in Table 1.

All patients had 24 h blood pressure measurement (ABPM). ABPM (Suntech Medical, USA, Morrisville, 2017) tests were performed between 4 and 6 days after the onset of a stroke. ABPM reading were recorded at 15 min intervals during the daytime and at 30 min intervals during the nighttime. Daytime and nighttime were defined as 6 a.m. to 10 p.m. and from 10 p.m. to 6 a.m., respectively. The recordings were analyzed using dedicated software and patients were excluded from the study if ≥20% of the measurements were not recorded successfully. Patients with mean nocturnal BP decline of ≥10% were defined as dippers, whereas those with a recorded decline < 10% were considered as nondippers [40]. The risk of OSAS has been assessed with the use of the Epworth sleepiness scale (ESS) whose sensitivity reaches up to 76%. The scale is useful as a screening tool and to control patients with OSAS that undergo therapy with the use of positive airway pressure [34]. The ESS was used for each situational question on a 4-point scale (from 0 to 3). Patients rated their likelihood of dozing off or falling asleep while engaged in eight usual activities. The ESS score was the sum of all eight situational sub-scores, with the total ranging from 0 to 24. The risk of OSAS increases with general score obtained by a patient. Severe excessive daytime OSAS can be recognized when the total score is >15 points [40].

### 2.2. Free Fatty Acids and Eicosanoid Detection

Fatty acid methyl esters were isolated from the serum with the use of the modified Folch and Szczuko methods [18,41]. The fatty acids profile was labeled by gas chromatography. The gas chromatography (GC) was performed with the use of the Agilent Technologies 7890A GC System (SUPELCOWAX™ 10 Capillary GC Column (15 mm × 0.10 mm, 0.10 μm); Supelco, Bellefonte, PA, USA). FAs were identified by comparing their retention times with those of Food Industry FAME Mix (Restek, Anchemplus, Poland).

The inflammatory metabolites were detected with the use of high-performance liquid chromatography (HPLC) (Agilent Technologies, UK). Among the analyzed mediators were: 9-HODE, 13-HODE, 5(S),6(R)-Lipoxin A_4_, 5(S),6(R),15(R)-Lipoxin A_4_, 5-HETE, 5-oxoETE, 12-HETE, 15-HETE, Leukotriene B4, Prostaglandin E2, Prostaglandin B2 16(R)/16(S)-HETE, 18-HEPE, 17-HDHA, 10(S)17(R)DiDHA (Protectin DX), Maresine1, and Rev D1, Rev E1. Detailed methodology was described elsewhere [18,42].

### 2.3. Statistical Analysis

Statistica 13.0 (Statsoft, Kraków, Poland) was used to perform the calculation dates. The assumptions for the use of parametric or non-parametric tests were checked using the Shapiro–Wilks test. Significant differences in mean values between the groups (group I—DIP and group II—NDIP) were assessed using one-way ANOVA and Tukey’s post hoc test. If the normality and homogeneity assumptions were violated, the Mann–Whitney non-parametric test was used. Statistical significance was set at *p* < 0.05. The FA’s matrix and derivatives were then correlated with the Epworth scale (ESS) with consideration of both groups (DIP, NDIP and all patients). The marked correlation coordinates are significant with *p* < 0.05.

## 3. Results

There was no association between dipping status and blood lipid levels (Table 1).

An increased level of C16:0 palmitic acid (saturated fatty acid) was observed in the nondipping compared to the dipping group, while a decrease was observed in regard to the level of other saturated fatty acids (C20:0 arachidic acid, C22:0 behenic acid) and a monounsaturated fatty acid (C24:1 nervonic acid) (Table 2). In comparisons between groups, the following tendency was observed with certain fatty acids: in the nondipping patients an elevated level of C20:3n3 cis-11-eicosatrienoic acid (eicosatrienoic acid, ETE, n3 PUFA), C22:4n6 docosatetraenoate acid (all-cis-4,7,10,13,16-docosapentaenoicacid, adrenic acid, n6 PUFA) and C22:2 cis-docosadienoic acid (docosadienoic acid, n6 PUFA) were recorded, while a decrease was observed in the level of C24:0 lignoceric acid (saturated fatty acid). The other fatty acids did not correlate with the dipping status (Table 2).

The HPLC analysis of the level of inflammatory mediators between the study groups showed that only the leukotriene B4 level was lower in the nondipping group (Table 3). There were no differences found in relation to the other tested inflammatory mediators.

The obtained results indicated that FA, pro and anti-inflammatory mediators in all analyzed patients showed no correlation with the ESS scale (Table S1). Only after introducing the division into the DIP and NDIP groups, the number of correlations with the ESS scale was found. The correlation between ESS and FAs in group I (DIP) showed a direct association with lowered C20: 0 arachidic acid, C22: 0 behenic acid, C22: 1n9 13 erucic acid, C22: 5w3 docosapentaenate, C24: 0 lignoceric acid and C24: 1 nervonic acid. Moreover, it was also shown that elevated levels of C15: 0 pentadecanoid acid, C17: 0 heptadecanoic acid, C22: 4n6 (docosatetraenoate) correlated with ESS. In the DIP group there was also a positive correlation of the ESS scale with eicosanoids such as prostaglandin E2, protectin D1, 17RS HDHA and negative correlation with 16RS HETE.

In group II, correlation between ESS and both FAs and eicosanoids showed a direct association with C14:1 myristolenic acid, C17:1 cis-10- heptadecanoid acid, C18:3n6 gamma linoleic acid, maresina 1, 18RS HEPE, 17RS HDHA, 5 oxo ETE. In the NDIP group, only the correlation between ESS and C18: 3n6 gamma linoleic acid was positive (Table S1).

## 4. Discussion

This is the first study to have shown possible pathogenetical connections between all the above-mentioned factors. We observed associations between the dipping status and the fatty acids in stroke patients. In the nondipping stroke subjects we detected lower levels of C24:1 nervonic acid (n9 MUFA), and diverse associations between SFAs and dipping status (Table 2). Palmitic acid is the most common SFA that can be provided by the diet and synthesized endogenously. Maintaining the proper ratio of n3 and n6 PUFA is crucial for keeping the membrane phospholipids in balance. The excessive accumulation of tissue palmitic acid results in hyperglycemia, fat accumulation, dyslipidemia and increased inflammation via Toll-like receptor 4 [43]. The palmitic acid level was found to be higher in patients with pathological, nondipping effect. This acid may play a role in cardiovascular disorders, inflammatory reactions and can increase the risk of stroke [6,8]. SFAs, including palmitic acid, were reported to be elevated in epicardial adipose tissue in patients with coronary artery disease [44]. The information regarding the role of arachidic acid is lacking, but we suggest that its decreased level in the nondipping group may result from its potential oxidation into a palmitic acid [45]. Another possible explanation involves the potential elongation of C14:0 myristic acid into the palmitic acid C16:0, because we detected lower levels of these acids in the NDIP group. Moreover, in our study myristolenic acids negatively correlated with the ESS.

We suggest that palmitic acid may act as an activator of inflammation in NDIP patients. A potential mechanism of proinflammatory activity can involve the Toll-like receptor 4 (TLR4) which is the main signaling pathway that triggers the obesity-induced inflammatory response. It is induced by the SFA and can be attenuated by the n3 PUFA by either lipopolysaccharides (LPS) or saturated fatty acids [46]. Further signaling processes lead to activation of transcription factor NF-κB that activates inflammatory cytokines such as Il-1, Il-6, Il-8 and TNF-α [35,47]. The anti-inflammatory effect of n-3 PUFA in the reduction in experimental brain damage due to hypoxia may be achieved by the effect in microglia by inhibiting NF-κB activation [48].

From the outcomes reported by other authors we conclude that the levels of SFAs including palmitic acid were associated with higher levels of low-density lipoprotein cholesterol (LDL-C), TC/high-density lipoprotein cholesterol (HDL-C) ratio, triglycerides, ApoB, ApoB/A1 ratio, hsCRP, and lower levels of HDL-C and ApoA1 [49]. No association was found between palmitic acid and coronary heart disease [50]. It seems reasonable that palmitic acid may be a significant proinflammatory factor that leads to the development of cardiovascular disorders, such as atrial fibrillation and heart failure [51]. Moreover, palmitic acid was positively associated with incident type 2 diabetes, but available data do not indicate a relationship between the very long chain saturated fatty acids such as arachidic acid and the risk of type 2 diabetes [52,53]. Contrary to palmitic acid, other SFAs such as arachidic and behenic acids were detected at lower levels in nondipping patients. There can be diverse mechanisms for their inflammatory effects or SFAs, such as arachidic acid and behenic acid, can be the substrates in the inflammatory process and thus their lower levels were observed in patients with the detrimental nondipping phenomenon. On the other hand, palmitic acid can have a harmful impact, while the other aforementioned SFAs can play a protective a role in the pathogenesis of stroke. The role of SFAs in the pathogenesis of stroke and in the inflammatory status is not yet unequivocal and needs further investigation, especially when taking into consideration the results of a recent meta-analysis that showed a protective role of SFAs in the risk of ischemic stroke [54].

Other fatty acids analyzed in our study including n3 and n6 as well as lipid levels did correlate with the dipping status. In particular, C18: 3n6 gamma linoleic acid in NDIP and the lower levels of C22: 0 behenic acid, C24: 0 lignoceric acid and C24: 1 nervonic acid in the DIP group are noteworthy, which correlated with the ESS scale (Table S1). Furthermore, the higher level of C22: 4n6 (docosatetraenoate) favored the occurrence of the dipping phenomenon with the ESS scale (Table S1). It was observed that, behenic acid and nervonic acid were decreased in metabolic syndrome and polycystic ovary syndrome (PCOS) patients, while a positive association was found in relation to HDL-C level and an inverse association with triglyceride levels [55,56].

A lower level of nervonic acid was found in the group of nondipping patients compared to dippers. Nervonic acid, which is a very long chain monounsaturated fatty acids (VLCFA), is an intermediate in the nerve cell myelin synthesis and is a component of membrane sphingolipids. The dietary interventions with this fatty acid are beneficial in the treatment of adrenoleukodystrophy [57,58]. Other authors showed that the level of nervonic acid is inversely correlated with LDL-C, HDL-C and directly associated with heart failure, cardiovascular risk, cardiovascular and all-cause mortality, markers of inflammation and endothelial activation (hsCRP, IL-6, ICAM-1) [59]. Nervonic acid is negatively connected with Il-1b level and IFN gamma, with no association with Il-6, Il-8, Il-10 [29]. Nervonic acid was also reduced along with a reduction in body weight in obese women and may have protective effects in obesity-related metabolic risk factors such as lipid levels, fasting blood glucose, CRP and leptin [60,61]. Lower levels of nervonic acid in patients with the nondipping phenomenon may indicate that it is potentially an inflammatory substrate and is used in the inflammatory cascade, as is the case with the SFAs. The VLCFAs may modulate the inflammation via the beta-oxidation in peroxisomes, which may lead to the synthesis of plasmalogens that are associated with oxidative stress and chronic inflammation [62]. The VLCFAs are elongated under the control of peroxisome proliferator-activated receptor (PPARα) leading to a decrease in nervonic acid level. Moreover, nervonic acid could be lower in nondipping stroke patients, not because of being a cause but a result of inflammation, because atherosclerosis may lower the peroxisomal activity and lead to degradation of VLCFAs [63].

**Figure 1:**
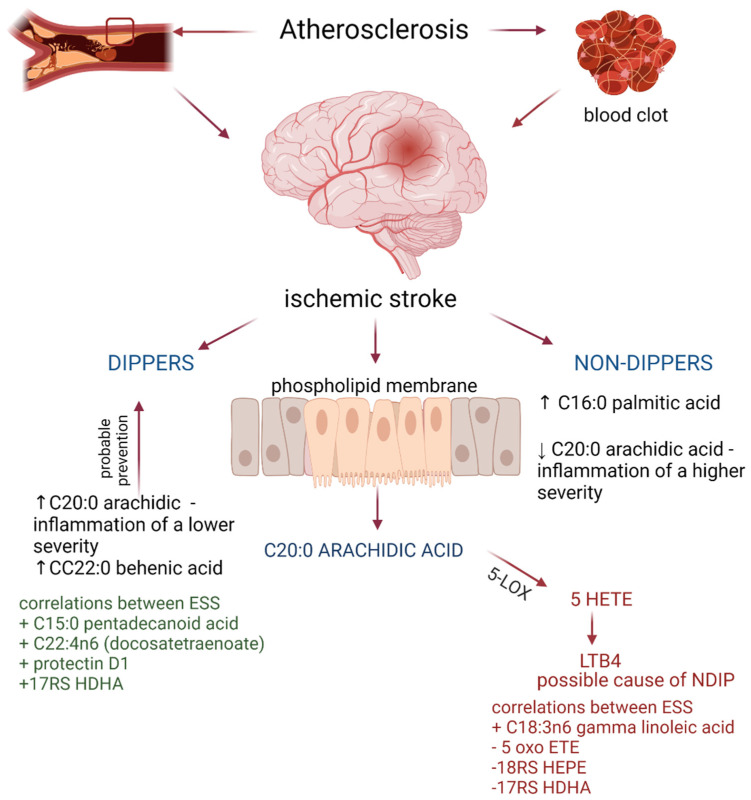
The participation of fatty acids and their mediators in the course of the DIP mechanism. (Created with BioRender.com https://app.biorender.com/, accessed on 8 August 2022). 5-LOX—lipooxygenase; 5HETE—5-hydroxyeicosatetraenoic acid; LTB_4_—leukotriene B; ESS—Epworth sleepiness scale.

In our study the leukotriene B4 level was lower in nondipping patients compared to the dipping group. On the other hand, low levels of oxo ETE and EPA and DHA derivatives (17RS HDHA, 18RS HEPE) were associated with the absence of the nondipping phenomenon. While the level of Protectin D1 and 17RS HDHA favored its occurrence in correlation with the ESS scale (Table S1). The n3 and n6 fatty acids affect the inflammation by cell membrane activation throughout the conversion by cyclooxygenases (COXs) and lipooxygenases (LOXs). The conversion products include prostaglandins (PGs), thromboxanes (TXs) and leukotrienes (LTs) [64]. Leukotrienes are synthesized in the inflammatory state especially in neutrophils and alveolar macrophages in the arachidonic cascade. Arachidonic acid is metabolized by 5-lipoxygenase to produce leukotrienes (LTB_4,_ LTC4, LTD4 and LTE4). Leukotrienes, in turn, play a role in the pathogenesis of chronic inflammation, oedema, leukocyte infiltration, while the level of 5-lipoxygenase correlates with the severity of atherosclerosis and atherosclerotic plaque instability. LTB4 activates the higher-affinity leukotriene receptor 1 (BLT1) which induces inflammation, enhances cytokine production and phagocytosis. Leukotriene B4 also activates the lower-affinity leukotriene receptor 2 (BLT2), but it is less known. Both receptors provoke NFκB activation and potentiate Toll-like receptor sterile inflammation. Therefore, LTB4 can play a central role in the development of metabolic diseases [65,66]. Taking into consideration the proinflammatory effects of LTB4, the explanation of its decreased level in nondipping stroke patients poses a real challenge. On the other hand, the direct association between LTB4 and ESS in the NDIP group may add to the pathogenetic importance of LTB4, because of the pathogenetic link between OSAS, inflammation and the nondipping phenomenon. As ESS is a tool for assessing the risk of OSAS, it is noteworthy that patients with OSAS have higher levels of LTB4. Additionally, the level of LTB4 was shown to be correlated with carotid atherosclerosis [67]. The role of leukotrienes in atherosclerosis development was documented at several levels, i.e., lipid retention and modification, intimal hyperplasia, endothelial dysfunction, atherosclerotic plaque formation, plaque rupture, myocardial and cerebral ischemia [68]. Nondipping patients have higher levels of arterial stiffness and cardiovascular risk [69]. The nondipping pattern has an additional negative effect on endothelial functions in hypertensive patients [63]. The inflammatory markers were shown to be increased in nondipping patients with OSAS (interleukin-2, CRP), while others were not different in such patients (IL-6, IL-8, IL-10, IL-12, and TNF-α) [31,32,70]. The nondipping status is not only related to proinflammatory but also to the procoagulant activity by increasing D-dimer, plasminogen activator inhibitor-1, von Willebrand factor, soluble intercellular adhesion molecule-1 and platelet-to-lymphocyte ratio [71,72]. The obtained results are shown in Figure 1.

We also demonstrated a direct association between MUFA acids (Table S1), anti-inflammatory protectin D1, 17RS HDHA and ESS score. Protectin D1 (10S,17S-dihydroxydocosahexaenoic acid;), 17RS HDHA and maresin D1 (7S,8R,17S-trihydroxy-4Z,9E,11E,13Z,15E,19Z-docosahexaenoic acid) belong to a family of specialized anti-inflammatory lipid mediators. These anti-inflammatory molecules are synthesized in the later stage of inflammation from EPA and DHA. The anti-inflammatory mediators play a role in the cardioprotective effects of n3 PUFAs beside the suppressive effect on arachidonic acid [73,74,75]. We detected a negative correlation between 16RS HETE and the ESS in stroke patients with the physiological dipping pattern. It means that EPA and DHA derivatives may have a beneficial effect in the pathogenesis of ESS and presumably, it could subsequently be involved in the protection against nondipping BP status. There are no such studies available to confront, but protectin D1 has anti-inflammatory properties, which allows us to justify our presumptions [76]. The role of ESS in cardiovascular disorders was elucidated in a recent study [77]. Such findings need further investigation because a potential link between OSAS and anti-inflammatory molecules in the pathogenesis of stroke and the nondipping phenomenon cannot be excluded. As with the anti-inflammatory properties of protectin D1 and resolving D1, MUFAs are supposed to function mainly as anti-inflammatory factors. They constitute an important ingredient of the Mediterranean diet. They inhibit the activation of NF-κB, NLR family pyrin domain containing 3 (NLRP3) and macrophages [78]. Higher intake of MUFAs is associated with lower pro-inflammatory (CRP, Il-6, I-18, IFN-γ, MCP-1, TNF-α) and elevated anti-inflammatory molecules (PPARγ, Il-4, Il-10) [79]. The role of anti-inflammatory lipids mediators and MUFAs in the risk of OSAS in the context of pathogenesis and the risk of stroke needs further studies. In our previous article, we demonstrated the effect of cyclooxygenase on the abolition of the thromboxane mediated dipping effect [80,81]. In the current study, we supplement the knowledge in terms of the participation of cyclooxygenase and the 5-LOX pathway with the participation of leukotriene.

The limitation of our study is the use of ESS as a screening test for detection of OSAS, while the results can also be influenced by other factors that change the quality of sleep. The classic definition and set hours of daytime and nighttime could be affected by the individual sleep-wake cycles connected with personal physiological differences and individual habits. When comparing the groups in the context of their characteristics, there was a higher prevalence of diagnosed diabetes in the dipping study patients. The observation is inconsistent with other studies which reported associations between diabetes and nondipping status [82]. We are also aware of the limited number of patients included in our study, but we suggest interpreting the presented results as a novel, pilot study requiring further analysis.

## 5. Conclusions

In our study we demonstrated the potential novel associations between the blood fatty acid levels and their inflammatory metabolites in the pathogenesis of the nondipping phenomenon in stroke patients. We suggest there are common connections between certain fatty acids, leukotriene B4, inflammation, hypertension, obstructive sleep apnea syndrome and nondipping profile. All these factors may play a role in the pathogenesis of stroke and need further investigation.

## Figures and Tables

**Table 1:** Characteristics of the study groups.

Parameter	Group I (DIP) Mean ± SD *n* = 33	Group II (NDIP) Mean ± SD *n* = 31	*p* Value
BMI (kg/m^2^)	29.13 ± 5.19	27.88 ± 4.17	NS
Age (years)	62.39 ± 11.95	58.45 ± 12.35	NS
Diabetes (*n*)	18/31	12/33	<0.05
Ischaemic heart disease (*n*)	4/31	2/33	NS
Hypertension (*n*)	24/31	22/33	NS
CRP (mg/L)	2.91 ± 5.11	2.14 ± 2.08	NS
TChol (mg/L)	204.48 ± 48.03	195.94 ± 57.36	NS
LDL (mg/L)	120.7 ± 41.23	112.45 ± 47.62	NS
HDL (mg/L)	54.36 ± 17.45	50.97 ± 14.11	NS
TG (mg/L)	151.82 ± 72.9	162.58 ± 83.58	NS

NS—not statistically significant; CRP—C-reactive protein; TChol—total cholesterol; LDL—low density lipoprotein; HDL—high density lipoprotein; TG—triglycerides.

**Table 2:** Comparison of fatty acids between group I (DIP) and II (NDIP).

FA [%]	Group I (DIP) *n* = 33	Group II (NDIP) *n* = 31	*p* Value
C13:0 tridecanoic acid	0.307 ± 0.1	0.31 ± 0.09	0.904
C14:0 myristic acid	1.265 ± 0.331	1.16 ± 0.435	0.277
C14:1 myristolenic acid	0.075 ± 0.036	0.066 ± 0.040	0.338
C15:0 pentadecanoic acid	0.221 ± 0.106	0.224 ± 0.124	0.925
C15:1 cis-10-pentadecanoic acid	0.078 ± 0.035	0.088 ± 0.04	0.295
C16:0 palmitic acid	26.294 ± 1.598	27.242 ± 1.649	0.023 *
C16:1 palmitoleic acid	2.261 ± 0.715	2.119 ± 0.791	0.453
C17:0 heptadecanoic acid	0.309 ± 0.045	0.294 ± 0.06	0.262
C17:1 cis-10-heptadecanoic acid	0.089 ± 0.038	0.098 ± 0.031	0.308
C18:0 stearic acid	13.216 ± 2.041	13.602 ± 1.948	0.443
C18:1n9 ct oleic acid	22.363 ± 4.108	22.117 ± 2.610	0.778
C18:1 vaccinic acid	1.996 ± 0.403	1965 ± 0.313	0.729
C18:2n6c linoleic acid	11.552 ± 2.328	11.757 ± 2.427	0.732
C18:2n6t linoleic acid	6.47 ± 1.784	5.865 ± 2.199	0.229
C18:3n6 gamma linoleic acid	0.417 ± 0.191	0.359 ± 0.165	0.202
C18:3n3 linolenic acid	0.524 ± 0.148	0.467 ± 0.139	0.119
C18:4 stearidonate	0.061 ± 0.026	0.052 ± 0.025	0.175
C20:0 arachidic acid	0.215 ± 0.054	0.187 ± 0.041	0.024 *
C22:1/C20:1 cis11-eicosanic acid	0.178 ± 0.064	0.171 ± 0.035	0.592
C20:2 cis-11-eicodienoic acid	0.152 ± 0.032	0.156 ± 0.037	0.581
C20:3n6 eicosatrienoic acid	1.347 ± 0.307	1.277 ± 0.321	0.378
C20:4n6 arachidonic acid	6.316 ± 1.355	6.364 ± 1.36	0.887
C20:3n3 cis-11-eicosatrienoic acid	0.029 ± 0.013	0.035 ± 0.016	0.062
C20:5n3 eicosapentaenoic acid	0.668 ± 0.301	0.574 ± 0.21	0.157
C22:0 behenic acid	0.245 ± 0.084	0.199 ± 0.08	0.028 *
C22:1n9 13 erucic acid	0.06 ± 0.018	0.061 ± 0.026	0.355
C22:2 cis-docodienoic acid	0.015 ± 0.011	0.02 ± 0.011	0.08
C23:0 tricosanoic acid	0.212 ± 0.092	0.23 ± 0.169	0.59
C22:4n6 docosatetraenoate	0.202 ± 0.099	0.26 ± 0.137	0.054
C22:5w3 docosapentaenate	0.442 ± 0.108	0.502 ± 0.339	0.348
C24:0 lignoceric acid	0.166 ± 0.068	0.135 ± 0.068	0.068
C22:6n3 docosahexaenoic acid	1.799 ± 0.591	1.713 ± 0.518	0.538
C24:1 nervonic acid	0.451 ± 0.24	0.328 ± 0.195	0.028 *

* statistically significant; FA—fatty acids; DIP—dipping effect; NDIP—non-dipping effect.

**Table 3:** Comparison of eicosanoids level between group I (DIP) and II (NDIP).

Eicosanoids [μg/mL]	Group I (DIP) *n* = 33	Group II (NDIP) *n* = 31	*p* Value
resolvin E1	0.043 ± 0.041	0.080 ± 0.131	0.120
prostaglandin E2	3.57 ± 5.248	2.701 ± 2.590	0.407
resolvin D1	0.180 ± 0.2	0.181 ± 0.328	0.971
** LTX A4 5S, 6R	0.00	0.041 ± 0.23	0.306
LTX A4 5S, 6R, 15R	0.027 ± 0.05	0.021 ± 0.037	0.53
protectin D1	0.047 ± 0.08	0.049 ± 0.049	0.938
maresin 1	0.032 ± 0.012	0.031 ± 0.019	0.61
leukotriene B4	0.031 ± 0.011	0.0225 ± 0.014	0.033 *
18RS HEPE	0.113 ± 0.039	0.104 ± 0.038	0.311
** 16 RS HETE	0.006 ± 0.039	0.00	0.337
13S HODE	0.037 ± 0.037	0.030 ± 0.022	0.317
9S HODE	0.039 ± 0.035	0.028 ± 0.017	0.122
15S HETE	0.309 ± 0.194	0.286 ± 0.242	0.678
17RS HDHA	0.115 ± 0.077	0.130 ± 0.08	0.435
12S HETE	1.918 ± 1.19	1.680 ± 1.138	0.418
5 oxo ETE	0.187 ± 0.084	0.184 ± 0.125	0.91
5 HETE	0.026 ± 0.009	0.025 ± 0.017	0.806

* statistically significant; LTX—lipoxin; HETE—hydroxyeicosatetraenoic acids; HODE—hydroxyoctadecadienoic acid; ** in only a few patients samples the concentration was at the limit of quantification.

## Data Availability

The data will be made available on request.

## Supplementary Materials

The following supporting information can be downloaded at: https://www.mdpi.com/article/10.3390/biomedicines10092200/s1. Table S1: Significant correlations between ESS, fatty acids and their inflammatory metabolites (separately for group I, group II).
