# Supplementary material for: Fatty Acid Levels and Their Inflammatory Metabolites Are Associated with the Nondipping Status and Risk of Obstructive Sleep Apnea Syndrome in Stroke Patients
Source: Biomedicines. 2022 Sep 6;10(9):2200. doi: 10.3390/biomedicines10092200 (PMC9496373; doi:10.3390/biomedicines10092200)
Supplement: Supplementary file 1 [file biomedicines-10-02200-s001.zip › biomedicines-1900143-supplementary.pdf]

Table S1. Significant correlations between ESS, fatty acids and their inflammatory metabolites (separately for group I, group II and all patients).

Parameter	Group I (DIP), r_s value	Group II (NDIP), r_s value	All patients r_s value
C13:0 Tridecanoic acid)	0.180	-0.050	0.078
C14:0 Myristic acid	0.069	-0.188	0.118
C14:1 Myristolenic acid	-0.079	-0.285*	0.036
C15:0 Pentadecanoid acid	0.454*	-0.133	0.187
C15:1 cis-10-pentadecanoid acid	0.218	-0.126	-0.005
C16:0 Palmitic acid	0.234	-0.117	-0.106
C16:1 Palmitoleic acid	0.054	0.058	0.098
C17:0 heptadecanoic acid	0.285*	-0.132	0.213
C17:1 cis-10- Heptadecanoid acid	0.206	-0.288*	-0.001
C18:0 Stearic acid	0.260	-0.010	0.057
C18:1n9 ct Oleic acid	-0.063	0.135	-0.004
C18:1 vaccinic acid	0.175	-0.189	0.118
C18:2n6c Linoleic acid	-0.256	-0.088	-0.159
C18:2n6t Linoleic acid	-0.184	-0.016	0.031
C18:3n6 gamma linoleic acid	0.058	0.375*	0.167
C18:3n3 linolenic acid	0.126	0.007	0.206
C18:4 (stearidonate)	-0.202	-0.266	0.011
C20:0 Arachidic acid	-0.421*	-0.097	-0.021
C22:1/C20:1 cis11- eicosanic acid	-0.164	-0.090	-0.051
C20:2 cis-11-eicodienoic acid	-0.172	-0.125	-0.137
C20:3n6 eicosatrienoic acid	-0.030	0.031	0.069
C20:4n6 Arachidonic acid	0.121	0.128	0.053
C20:3n3 cis-11-eicosatrienoic acid	-0.091	-0.220	-0.223
C20:5n3 EPA	0.044	0.199	0.164
C22:0 Behenic acid	-0.354*	0.104	0.035
C22:1n9 13 Erucic acid	-0.282*	-0.168	-0.058
C22:1cis13	-0.279	0.048	-0.195
C22:2 cis-docodienoic acid	-0.138	-0.055	-0.229
C23:0 tricosanoic acid	0.082	-0.173	-0.035
C22:4n6 (docosatetraenoate)	0.456*	-0.016	0.002
C22:5w3 (docosapentaenate)	-0.281*	0.027	-0.144
C24:0 Lignoceric acid	-0.335*	0.141	0.016
C22:6n3 DHA	0.064	-0.010	0.090
C24:1 Nervonic acid	-0.381*	0.012	0.006
Resolvina E1	0.108	-0.129	-0.131
Prostaglandin E2	0.289*	-0.145	0.223
Resolvina D1	0.149	-0.051	0.047
LTX A4 5S, 6R	**	-0.171	-0.109
LTX A4 5S, 6R, 15R	0.204	-0.048	0.167
Protectin D1	0.341*	-0.214	0.184
Maresina 1	0.156	-0.295*	0.084
Leucotriene B4	-0.066	-0.153	0.158
18RS HEPE	0.087	-0.445*	0.111
16RS HETE	-0.302*	**	-0.114
13S HODE	0.223	-0.199	0.215

9S HODE	0.098	-0.223	0.194
15S HETE	0.131	-0.223	0.081
17RS HDHA	0.527*	-0.415*	0.153
12S HETE	0.053	-0.179	0.092
5 oxo ETE	0.196	-0.448*	0.057
5 HETE	0.118	-0.231	0.044

**the marked correlation coordinates are significant with p <0.05; ** only a few patients samples the concentration was at the limit of quantification*
